# Comparison of decision tree with common machine learning models for prediction of biguanide and sulfonylurea poisoning in the United States: an analysis of the National Poison Data System

**DOI:** 10.1186/s12911-022-02095-y

**Published:** 2023-04-06

**Authors:** Omid Mehrpour, Farhad Saeedi, Samaneh Nakhaee, Farbod Tavakkoli Khomeini, Ali Hadianfar, Alireza Amirabadizadeh, Christopher Hoyte

**Affiliations:** 1 Data Science Institute, Southern Methodist University, Dallas, TX USA; 2 Medical Toxicology and Drug Abuse Research Center (MTDRC), Birjand University of Medical Sciences, Birjand, Iran; 3 Student Research Committee, Birjand University of Medical Sciences, Birjand, Iran; 4 Department of Epidemiology and Biostatistics, Mashhad University of Medical Sciences, Mashhad, Iran; 5 Endocrine Research Center, Research Institute for Endocrine Sciences, Shahid Beheshti University of Medical Sciences, Tehran, Iran; 6 University of Colorado Anschutz Medical Campus, Aurora, CO USA

**Keywords:** Biguanide, Sulfonylurea, Overdose, Decision tree, National Poison Data System, NPDS

## Abstract

**Background:**

Biguanides and sulfonylurea are two classes of anti-diabetic medications that have commonly been prescribed all around the world. Diagnosis of biguanide and sulfonylurea exposures is based on history taking and physical examination; thus, physicians might misdiagnose these two different clinical settings. We aimed to conduct a study to develop a model based on decision tree analysis to help physicians better diagnose these poisoning cases.

**Methods:**

The National Poison Data System was used for this six-year retrospective cohort study.The decision tree model, common machine learning models multi layers perceptron, stochastic gradient descent (SGD), Adaboosting classiefier, linear support vector machine and ensembling methods including bagging, voting and stacking methods were used. The confusion matrix, precision, recall, specificity, f1-score, and accuracy were reported to evaluate the model’s performance.

**Results:**

Of 6183 participants, 3336 patients (54.0%) were identified as biguanides exposures, and the remaining were those with sulfonylureas exposures. The decision tree model showed that the most important clinical findings defining biguanide and sulfonylurea exposures were hypoglycemia, abdominal pain, acidosis, diaphoresis, tremor, vomiting, diarrhea, age, and reasons for exposure. The specificity, precision, recall, f1-score, and accuracy of all models were greater than 86%, 89%, 88%, and 88%, respectively. The lowest values belong to SGD model. The decision tree model has a sensitivity (recall) of 93.3%, specificity of 92.8%, precision of 93.4%, f1_score of 93.3%, and accuracy of 93.3%.

**Conclusion:**

Our results indicated that machine learning methods including decision tree and ensembling methods provide a precise prediction model to diagnose biguanides and sulfonylureas exposure.

## Introduction

Sulfonylureas and biguanides are two types of anti-diabetic medications widely prescribed in the United States [1]. Some of the biguanides medications are buformin, phenformin, and metformin. Phenformin has not been used in the United States or Europe since 1978 due to concerns about lactic acidosis, while metformin is still available to treat diabetes [2, 3]. According to the US National Poison Data System (NPDS), exposure to oral anti-diabetic medications increased by 35% between 2001 and 2004 [4–6]. Based on the NPDS report in 2015, 3837 and 8733 exposures to sulfonylureas and biguanides were reported to the poison control centers, respectively. The majority of them were unintentional [7]. In 2007, sulfonylureas overdoses accounted for 34% of all oral hypoglycemic and antihyperglycemic medication overdoses [8]. The mortality rate of biguanides overdose (6.1%) is greater than that of sulfonylureas overdoses (3.6%) [9]. Sulfonylureas poisoning gives rise to symptoms including dizziness, weakness, headache, confusion, lethargy, slurred speech, coma, seizures, tachycardia, palpitations, nausea, and diaphoresis [10]. Abdominal pain, vomiting and diarrhea, altered mental state, lactic acidosis, hypotension, and arrhythmia are the presentations of biguanide overdose [11, 12]. Anti-diabetic medication overdoses, including biguanides or sulfonylureas poisoning, are a common cause of hypoglycemia; however, hypoglycemia caused by sulfonylurea overdose is more prevalent than biguanides overdose [13]. The overdose of these medications may cause serious morbidity and necessitate extensive and prolonged medical treatment. Early therapy reduces the risk of fatalities and permanent consequences [6, 14]. The toxicity of oral antidiabetic agents differs widely in clinical manifestations, severity, and treatment [6, 15]. It is important for the emergency physician to identify the drug class to which the patient may have been exposed during blood glucose stabilization and assessment in order to predict complications and make appropriate decisions [14]. The management of the sulfonylureas focuses primarily on restoring and maintaining euglycemia. The greatest risk associated with antihyperglycemic agents is with regards to metformin. With this medication, hypoglycemia is not a major concern; the primary concerns are cardiovascular collapse and renal failure caused by profound lactic acidosis. The treatment of these adverse events focuses on restoring the acid-base balance through the use of sodium bicarbonate and hemodialysis [6, 14].

Since the diagnosis of biguanides and sulfonylureas overdoses is based on history taking and physical examination, it is crucial to develop an algorithm to help physicians make a better diagnosis. Although these drugs might be detected in urine analysis, this method is not commonly employed in facilities with limited resources. So, designing a clinical decision algorithm for distinguishing exposure to these pharmaceutical drugs is crucial. Clinical decision analysis is a powerful tool for addressing complexity and uncertainty in medical problems by utilizing evidence-based medicine [16]. Machine learning (ML) can be used to make diagnoses, determine treatment decisions, and predict outcomes [17].

Due to its superior classification accuracy and simple representation of collected data, the decision tree (DT) is one of the most commonly used machine learning methods in a wide range of medical situations requiring consistent decision-making [18]. A growing body of studies has demonstrated the effectiveness of decision tree analysis in disease diagnosis [19–22]. A robust classification tool is provided by this model. This approach proposes an understandable model based on current observations using a simple technique. The model proposed by the structure is both understandable and accessible [23]. The decision tree has the following advantages: 1- It can be visualized and is simple to understand and interpret. It requires very little data preparation compared to other techniques which often require the normalization of data, the creation of dummy variables and the removal of blank values. In addition, the cost associated with using the tree (for predicting data) is directly proportional to the number of data points used to train the tree. In contrast to other techniques, decision trees are capable of handling both categorical and numerical data. Other techniques are specialized for only one type of variable. There is no limit to the number of outputs that can be handled by decision trees. This model utilizes a white box model, which means that there are usually two outputs, which can be explained easily by Boolean logic. For instance, yes or no [24]. In some studies, data mining and statistical approaches have been compared in order to solve prediction problems. In these comparison studies, data sets or distributions of dependent variables have been mainly considered. A comparison of logistic regression and decision trees was carried out by Long et al. Both the LR tool and the improved trees were found to perform at a level similar to that of the physicians [25]. A number of different methods are examined by Li and Jain for the classification of documents. These methods include naive Bayes classifiers, nearest neighbour classifiers, decision trees, and a subspace method. Experimental results indicate that all four classification algorithms perform reasonably well [26]. In medical toxicology, few studies to date have used some machine learning algorithms on national poisoning data to identify the potential cause of the poisoning [27–31] and to our knowledge there is no study on prediction of patients with anti-diabetic exposure using decision tree model. Given the growing utilization of decision trees in disease diagnosis and the concern over biguanides and sulfonylureas overdose misdiagnosis, we aimed to conduct a study to develop a model based on decision tree analysis to help physicians better diagnose biguanide and sulfonylurea poisoning.

## Materials and methods

### Study design and population

The data of this observational study on the general population was obtained from the NPDS. The American Association of Poison Control Centers (AAPCC) maintains that the National Poison Data System (NPDS) contains de-identified case records of self-reported information collected from callers during exposure management and poison information calls managed by the 55 country’s poison control centers across the United States. NPDS data do not reflect the entire universe of exposure to a particular substance, as additional exposure to PCCs may be unreported. Therefore, NPDS data should not be interpreted as the total incidence of US exposure to any substance (s). In addition, exposures do not necessarily represent a poisoning or overdose, and AAPCC cannot fully verify each report’s accuracy. Therefore, results based on NPDS data do not necessarily reflect the opinions of the AAPCC. The inclusion criteria were any exposure to biguanides and sulfonylureas between January 1, 2012, and December 31, 2017, reported to the poison control centers across the United States. Exclusion criteria were missing demographic data or irrelevant medical outcomes. Sex, the reason for exposure, age, exposure’s signs, and symptoms were among the variables obtained for analysis and evaluation of basic characteristics. Hence, the study size was determined based on the number of participants meeting eligibility criteria. We defined reasons for exposure as following: intentional exposure, unintentional exposure, and others. Medical outcomes were classified as minor, moderate, and major, and were assessed by expert medical toxicologist blindly. Full definitions of clinical features can be found in the NPDS coding manual version 3.1 [29, 32]. The outcome that is predicted by the prediction model was sulfonylureas and biguanides poisoning. Regarding the guidelines and policies of the Colorado Multiple Institutional Review Board on Human Subjects Protection, the analysis of NPDS data for this research study does not meet the criteria for human subjects under the 45 Code of Federal Regulations (CFR) 45 CFR 46.101(b) and hence, no approval of the institutional review board was required. This study was reviewed by Colorado Multiple Institutional Review Board on Human Subjects Protection and determined to be exempt (COMIRB#: 22-1088).

#### Pre processing

We used Recursive Feauture Elimination (RFECV) to select optimal features, then we standardize the data and used 10 folds cross validation to minimize the overfitting risk and finally generated the classification report.

#### Decision tree development and evaluation

In recent years, the decision tree has found itself in medicine to offer suggestions to decide the medical diagnosis, treatment, and prognosis. The mechanism by which a decision tree functions is based on some IF-THEN rules, meaning that the outcomes are illustrated through some conditions. Decision trees are a kind of non parametric models that can be used for both classification and regression. They can either output a categorical prediction or a numerical prediction. They classify instances by sorting them down from the root to some leaf nodes. The ease of interpretation is one of the primary benefits of decision trees. Decision trees provide the results in a graphical and tree-shaped diagram. They require less training data than other machine learning algorithms. And they are tolerant to missing values. Every decision tree model applies the rules down the path from the root node, which is the first node of the model, to the leaf nodes, which are the outcomes that the model considers following a decision-making process. In our model, right and left directions represent that the rules are true and false, respectively. We utilized recall, specificity, f1-score, accuracy, confusion matrix, and precision in evaluation. Recall means that the true positive proportion is divided into the number of positive events regardless of whether they are predicted correctly. Precision means the proportion of the true positive divided by the total positive prediction in our sample. Accuracy means how many predictions are correctly made by our model. F1-score represents a weighted proportion of recall and precision. Lastly, the confusion matrix is regarded as a visually appealing method of assessing the model’s performance.

#### Comparative analysis

In order to compare decision tree with other machine learning models, we applied stochastic gradient descent (SGD), gradient boosting classification, multi layers perceptron (MLP),

Adaboosting classiefier, linear support vector machine (SVM_linear), gradient boosting, light gradient boosting, voting, bagging and stacking ensembling to our datasets. After 10-fold cross validation, the metrics for each model were reported.

### Statistical analysis

To assess the normality of the quantitative variables, we employed the Kolmogorov–Smirnov test. Student’s t-test or Mann–Whitney U and the chi-squared test was used to compare the two groups.The analysis of the data was performed with python 3.7. A P-value < 0.05 was considered statistically significant.

## Results

**Fig. 1:**
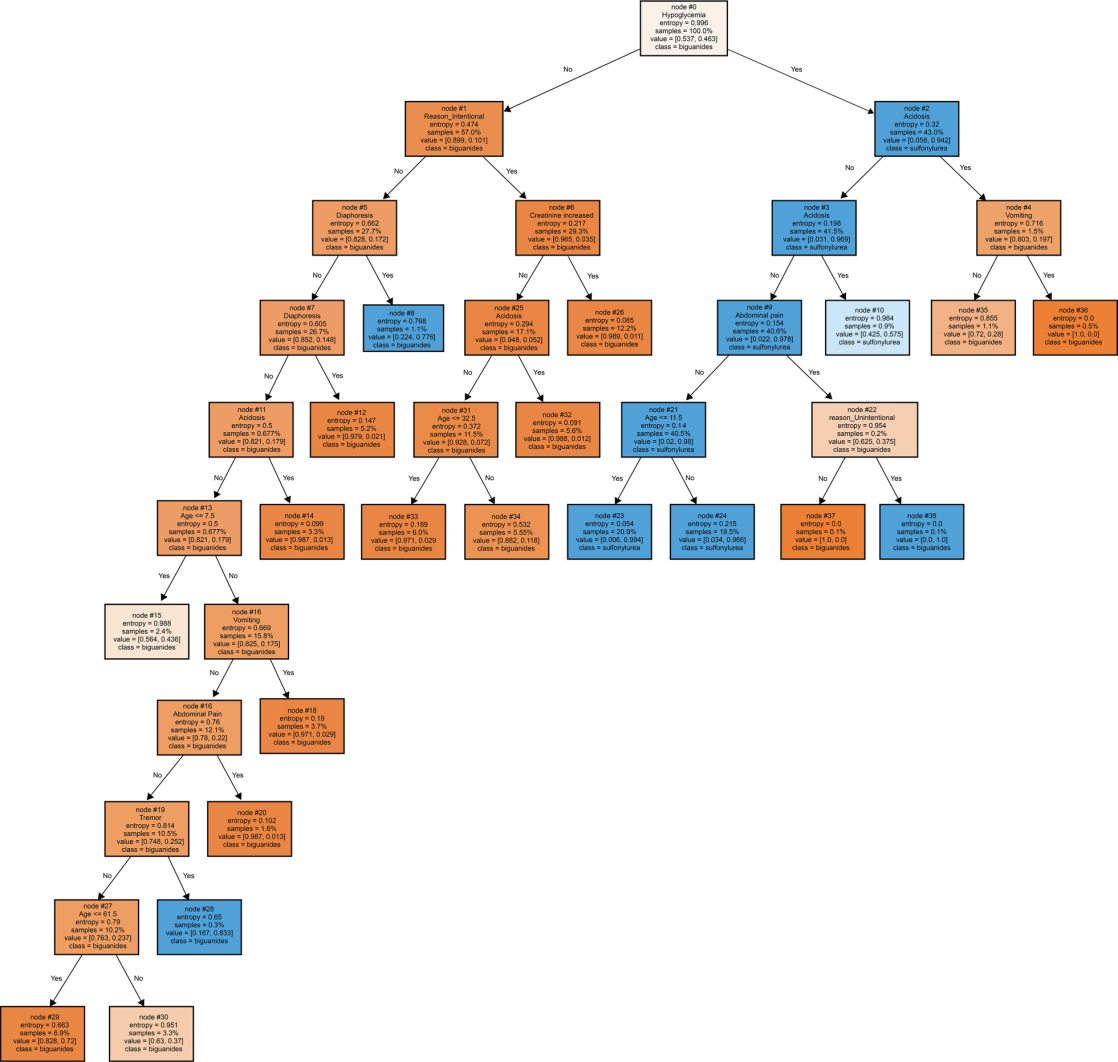
Decision tree model with training data. Values are the percentages of (Biguanides exposure, sulfonylureas exposure). The blue color indicates sulfonylurea, while the orange color shows biguanide exposure

Among the 6183 participants, 3336 patients (54.0%) were identified as biguanides exposure cases, and the remaining were the ones with sulfonylureas exposures. This populationcomprised of 3706 females (59.9%) and 2477 males (40.1%). Intentional and unintentional exposures were found in 2246 (36.3%) and 3199 (51.7%) cases, respectively. Minor, moderate, and major outcomes were reported in 2461 (39.8%), 3244 (52.4%), and 419 (6.8%) cases, respectively (Table 1). The size of the decision tree developed in this study was 39, which included 20 leaves and 11 layers (Fig. 1). Hypoglycemia was the root node of our model.

The rules derived from the decision tree are shown in Table 2. The characteristics of machine learning models used for comparative analysis are shown in Table 3. The specificity, precision, recall, f1-score, and accuracy of all models were greater than 86%, 89%, 88%, and 88%, respectively. The lowest values belong to SGD model. The decision tree model has a sensitivity (recall) of 93.3%, specificity of 92.8%, precision of 93.4%, f1_score of 93.3%, and accuracy of 93.3%.

**Table 1:** Baseline characteristics of study participants

Variables	Biguanides	Sulfonylureas	p-value
*Sex*			
Male	1127 (33.8)	1350 (47.4)	< 0.001
Female	2209 (66.2)	1497 (52.6)	
*Reason of exposure*			
Intentional			
Yes	1828 (54.8)	418 (14.7)	< 0.001
No	1508 (45.2)	2429 (85.3)	
Unintentional			
Yes	1189 (35.6)	2010 (70.6)	< 0.001
No	2147 (64.4)	837 (29.4)	
Other			
Yes	64 (1.9)	83 (2.9)	0.01
No	3272 (98.1)	2764 (97.1)	
*Medical outcomes*			
Minor			
Yes	2012 (60.3)	449 (15.8)	< 0.001
No	1324 (39.7)	2398 (84.2)	
Moderate			
Yes	1063 (31.9)	2181 (76.6)	< 0.001
No	2273 (68.1)	666 (23.4)	
Major			
Yes	210 (6.3)	209 (7.3)	0.10
No	3126 (93.7)	2638 (92.7)

**Table 2:** The 20 rules extracted through the decision tree

1	IF there is hypoglycemia, acidosis, and vomiting, THEN the patients are poisoned by biguanides (100%)
2	IF there is hypoglycemia and acidosis, vomiting is not present; THEN the patients are more likely to be poisoned by biguanides (72%)
3	IF hypoglycemia and vomiting are present, without acidosis, THEN the patients are more likely to be poisoned by sulfonylureas (57.5%)
4	If hypoglycemia and abdominal pain are present, acidosis and vomiting are not present, the reason for exposure is unintentional, THEN the patients are poisoned by sulfonylureas (100%)
5	If hypoglycemia and abdominal pain are present, acidosis and vomiting are not present, the reason for exposure is not unintentional, THEN the patients are poisoned by biguanides (100%)
6	If hypoglycemia is present, acidosis, vomiting, abdominal pain are not present, and the age is greater than 11.5 years, THEN the patients are poisoned by sulfonylureas (96.6%)
7	If hypoglycemia is present, acidosis, vomiting, and abdominal pain are not present, and the age is less than 11.5 years, THEN the patients are poisoned by sulfonylureas (99.4%)
8	If hypoglycemia is not present, the reason for exposure is intentional, and vomiting is present, THEN the patients are more likely to be poisoned by biguanides (98.9%)
9	IF hypoglycemia and vomiting are not present, the reason for exposure is intentional, and acidosis is present, THEN the patients are more likely to be poisoned by biguanides (98.8%)
10	IF hypoglycemia, acidosis, and vomiting are not present, the reason for exposure is intentional, and age is greater than 32.5 years, THEN the patients are more likely to be poisoned by biguanides (88.2%)
11	IF hypoglycemia, acidosis, and vomiting are not present, the reason for exposure is intentional, and age is less than 32.5 years, THEN the patients are more likely to be poisoned by biguanides (97.1%)
12	If hypoglycemia is not present, the reason for exposure is not intentional, and diaphoresis is present, THEN the patients are more likely to be poisoned by sulfonylureas (77.6%)
13	IF hypoglycemia and diaphoresis are not present, the reason for exposure is not intentional, and diarrhea is present, THEN the patients are more likely to be poisoned by biguanides (97.9%)
14	IF hypoglycemia, diaphoresis, and diarrhea are not present, the reason for exposure is not intentional, and acidosis is present, THEN the patients are more likely to be poisoned by biguanides (98.7%)
15	IF hypoglycemia, diaphoresis, diarrhea, and acidosis are not present, the reason for exposure is not intentional, and age is less than 7.5 years, THEN the patients are more likely to be poisoned by biguanides (56.4%)
16	IF hypoglycemia, diaphoresis, diarrhea, and acidosis are not present, the reason for exposure is not intentional, and age is greater than 7.5 years old, vomiting is present, THEN the patients are more likely to be poisoned by biguanides (97.1%)
17	IF hypoglycemia, diaphoresis, diarrhea, vomiting, and acidosis are not present, the reason for exposure is not intentional, and age is greater than 7.5 years old, abdominal pain is present, THEN the patients are more likely to be poisoned by biguanides (98.7%)
18	IF hypoglycemia, diaphoresis, diarrhea, vomiting, abdominal pain, and acidosis are not present, the reason for exposure is not intentional, and age is greater than 7.5 years old, tremor is present, THEN the patients are more likely to be poisoned by sulfonylureas (83.3%)
19	IF hypoglycemia, diaphoresis, diarrhea, vomiting, abdominal pain, tremor, and acidosis are not present, the reason for exposure is not intentional, and age is greater than 61.5 years, THEN the patients are more likely to be poisoned by biguanides (63%)
20	IF hypoglycemia, diaphoresis, diarrhea, vomiting, abdominal pain, tremor, and acidosis are not present, the reason for exposure is not intentional, and age is between 7.5–61.5 years old, THEN the patients are more likely to be poisoned by biguanides (82.8%)

**Table 3:** Characteristics of ML models used in comparative analysis

Labels	ML models	Biguanides	Sulfonylurea	Average	Weighted_average
Specificity	Adaboosting	0.907973	0.954436	0.931205	0.929368
	DT	0.898138	0.963429	0.930784	0.928202
	SGD	0.790657	0.960731	0.875694	0.868969
	SVM_linear	0.896382	0.962230	0.929306	0.926702
	MLP	0.913593	0.952338	0.932966	0.931434
	Gradient boosting	0.901300	0.969125	0.935212	0.932530
	Light gradient boosting	0.909730	0.954736	0.932233	0.930453
	Voting-ensemble	0.895328	0.972422	0.933875	0.930827
	Bagging ensemble	0.892870	0.973022	0.932946	0.929776
	Stacking ensemble	0.893572	0.973321	0.933447	0.930293
Precision	Adaboosting	0.923970	0.944465	0.934217	0.933407
	DT	0.917237	0.954461	0.935849	0.934377
	SGD	0.843199	0.945004	0.894102	0.890076
	SVM_linear	0.915835	0.952950	0.934392	0.932925
	MLP	0.928133	0.942391	0.935262	0.934698
	Gradient boosting	0.920034	0.961409	0.940721	0.939085
	Light gradient boosting	0.925334	0.944911	0.935122	0.934348
	Voting-ensemble	0.915867	0.965165	0.940516	0.938566
	Bagging ensemble	0.914109	0.965805	0.939957	0.937913
	Stacking ensemble	0.914648	0.966198	0.940423	0.938385
Recall	Adaboosting	0.954436	0.907973	0.931205	0.933042
	DT	0.963429	0.898138	0.930784	0.933366
	SGD	0.960731	0.790657	0.875694	0.882420
	SVM_linear	0.962230	0.896382	0.929306	0.931910
	MLP	0.952338	0.913593	0.932966	0.934498
	Gradient boosting	0.969125	0.901300	0.935212	0.937894
	Light gradient boosting	0.954736	0.909730	0.932233	0.934013
	Voting-ensemble	0.972422	0.895328	0.933875	0.936924
	Bagging ensemble	0.973022	0.892870	0.932946	0.936115
	Stacking ensemble	0.973321	0.893572	0.933447	0.936600
F1_score	Adaboosting	0.938956	0.925860	0.932408	0.932926
	DT	0.939766	0.925443	0.932605	0.933171
	SGD	0.898136	0.860968	0.879552	0.881022
	SVM_linear	0.938459	0.923801	0.931130	0.931710
	MLP	0.940080	0.927769	0.933924	0.934411
	Gradient boosting	0.943942	0.930384	0.937163	0.937699
	Light gradient boosting	0.939805	0.926986	0.933396	0.933903
	Voting-ensemble	0.943297	0.928936	0.936117	0.936685
	Bagging ensemble	0.942646	0.927907	0.935276	0.935859
	Stacking ensemble	0.943073	0.928467	0.935770	0.936348
Accuracy	Adaboosting	–	–	0.933042	0.933042
	DT	–	–	0.933366	0.933366
	SGD	–	–	0.882420	0.882420
	SVM_linear	–	–	0.931910	0.931910
	MLP	–	–	0.934498	0.934498
	Gradient boosting	–	–	0.937894	0.937894
	Light gradient boosting	–	–	0.934013	0.934013
	Voting-ensemble	–	–	0.936924	0.936924
	Bagging ensemble	–	–	0.936115	0.936115
	Stacking ensemble	–	–	0.936600	0.936600

*DT* Decision tree, *MLP* Multi layers perceptron, *SGD* Stochastic gradient descent, Adaboosting classiefier, SVM_linear: linear support vector machine

**Fig. 2:**
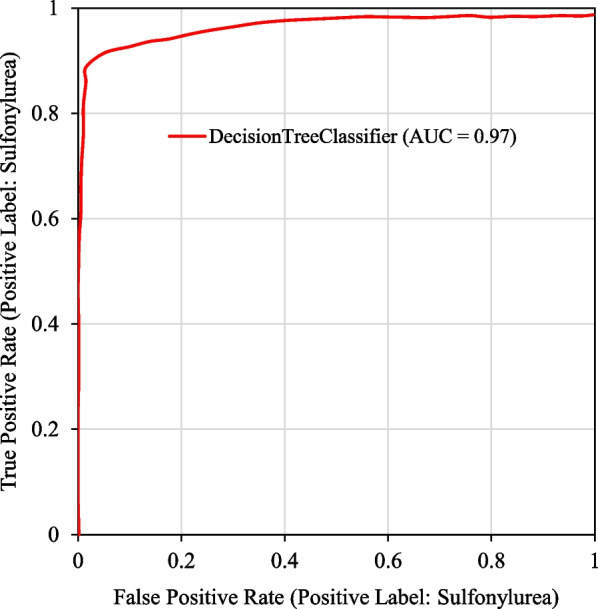
The Area under the Curve (AUC) graph of the decision tree

**Fig. 3:**
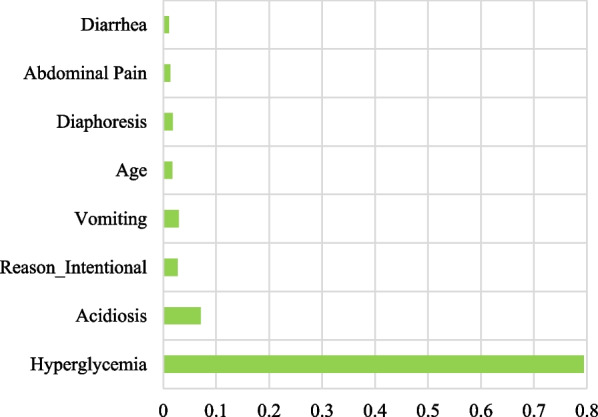
Important features in the diagnosis of antihyperglycemic exposure based on decision tree model

The confusion matrices of training and test datasets is shown in Table 4. The Stacking ensemble and MLP have lowest false detections assigned to Biguanides and Sulfonylurea, respectively. The area under the curve for decision tree model was 0.97 (Fig. 2). In Fig. 3, we evaluated important clinical findings that affect the DT’s performance (i.e., feature importance) in the classification task. A few features like ‘hypoglycemia’ and ‘acidosis’ contributed most to classifying two products, respectively. The feature importance implicitly indicates that if we look at only these few features and combine their presence in a test case, we might identify a poisoning/exposure to a specific product.

**Table 4:** Confusion matrice of ML models used in comparative analysis

Prediction true	Models	Biguanides	Sulfonylurea
Biguanides	Ada	3184	152
	DT	3214	122
	SGD	3205	131
	SVM_linear	3210	126
	MLP	3177	159
	Gradient Boosting	3233	103
	Light Gradient Boosting	3185	151
	Voting-ensemble	3244	92
	Bagging ensemble	3246	90
	Stacking ensemble	3247	89
Sulfonylurea	Ada	262	2585
	DT	290	2557
	SGD	596	2251
	SVM_linear	295	2552
	MLP	246	2601
	Gradient Boosting	281	2566
	Light Gradient Boosting	257	2590
	Voting-ensemble	298	2549
	Bagging ensemble	305	2542
	Stacking ensemble	303	2544

*DT* Decision tree, *MLP* Multi layers perceptron, *SGD* Stochastic gradient descent, Adaboosting classiefier, SVM_linear: linear support vector machine

## Discussion

Biguanide and sulfonylureas are two popular antihyperglycemic agents commonly used by diabetic patients. In addition, antihyperglycemic side effects or overdoses are also common in these patients. Sometimes the clinical symptoms of these drugs are close, which leads to misdiagnosis. So correct diagnosis at an early stage is critical for treatment and management. This study proposed an accurate decision tree prediction model for biguanide and sulfonylurea poisoning diagnoses using a retrospective analysis of large-scale NPDS data. This study found that the decision tree is a reliable algorithm for identifying and distinguishing antihyperglycemic agents. Also, hypoglycemia, abdominal pain, acidosis, diaphoresis, tremor, vomiting, diarrhea, age, and reasons for exposure were critical for making the diagnosis.

There are three means of learning: experience, which is the most challenging; imitation, which is the simplest; and thinking, which is the most complex [33]. Algorithmic thinking in medicine for the prediction of the prognosis or diagnosis of a disease or the selection of appropriate treatment are very helpful. Typically, this type of thinking arises from the analysis of prospective clinical studies data, which can be done by a human, or using biostatistical models, or both. Doctors are learned to use algorithmic thinking in the following manner: they check for the symptoms, signs, or risk factors indicated, they add the points, and they obtain a diagnostic or prognostic probability of the presence of the disease. It is difficult to diagnose many diseases, and their clinical symptoms are often hard to identify. Fortunately, many medical algorithms are validated. Algorithms are frequently used by emergency physicians. They are particularly fond of scoring systems. In addition, the knowledge of algorithms, both practical and theoretical, allows physicians to better understand their limitations. Furthermore, algorithmic thinking has become widespread in medical knowledge as a result. As such, physicians are intrigued by algorithmic thinking when diagnosing disease, predicting prognosis, or choosing appropriate treatment. Many decision trees are suggested in medical textbooks. As a result, decision tree algorithms found in ML algorithms have the potential to attract physicians’ attention. Thus, the DT does not only assist physicians in diagnosing a disease or predicting the outcome, but also assists them in organizing algorithmic reasoning. In recent years, predictive models have been employed for disease diagnosis [34]. The decision tree model is a data mining algorithm for disease prediction by employing multiple variables and risk factors [35]. In addition, decision tree analysis is a prominent approach for dealing with non-linear relationships and developing feasible and clear rules [36–38]. It is a non-parametric modeling algorithm and can fit any type of functional forms. In addition, it uses a recursive binary partitioning algorithm that divides the sample into partitions with the strongest association with the response variable based on the partitioning variable [39]. Therefore, this model is a robust classification tool. Furthermore, this approach suggests a comprehensible model for the current observations using a simple technique. Therefore, it suggests a model structure that is understandable and accessible [40]. Some documents have shown that predicting models can identify diseases with an accuracy similar to that of human specialists [41–45]. In general, the prediction algorithms may not go beyond human judgment; instead, they can be a powerful auxiliary tool to circumvent when used properly by trained physicians [46]. Prediction models can help in clinical decisions making but it will not replace the physician completely. In medicine, human errors are associated with large financial problems, and many of them can be prevented with the help of these models [47]. The first line who benefits of DT could be specialists in poisoning information (SPIs). Other clinicians who might encounter poisoned patients during their practice would also be targeted. Several of them lacked medical toxicology training, so the system would be helpful to them. It is important to note, however, that not all patients present to the emergency room with a classic symptom. Furthermore, some centers lack laboratory facilities. As a result, even the medical toxicologist will benefit from this system. We also achieved a very high specificity, which makes it easier for medical toxicologists to confirm their initial diagnosis. Therefore, this system can also be utilized as a confirmation method.

In our decision tree model, the most relevant clinical finding and the essential variable in predicting was hypoglycemia, selected as the tree’s root node. Hypoglycemia is the most common side effect of sulfonylureas overdose [2]. Biguanides can modify blood glucose levels in diabetic individuals but not in non-diabetic persons [48]. Bron et al. conducted a study on over 200,000 type-2 diabetes mellitus patients who were on anti-diabetic medications to evaluate the risk of hypoglycemia and found that sulfonylureas were more likely to cause hypoglycemia than biguanides, such as metformin [49]; unlike sulfonylurea, hypoglycemia rarely occurs in biguanides poisoning [50, 51]. Moreover, it has been noted that metformin-induced hypoglycemia has an incidence ranging from 0.6 to 12.2% [52]. Hypoglycemia in biguanides exposure is not common because they increase insulin sensitivity and does not increase insulin release. In contrast, hypoglycemia following sulfonylureas exposure would be more common due to neuroglycopenic effects and counterregulatory hormonal response [13]. Besides, sulfonylureas could increase insulin release from pancreatic beta cells by acting on ATP-sensitive potassium channels, leading to hypoglycemia [53]. Sulfonylurea-induced hypoglycemia depletes ATP levels in the central nervous system, which can lead to clinical manifestations such as dizziness or vertigo, tremors, restlessness, drowsiness, or lethargy, as well as hormonal counterregulatory responses like diaphoresis [13]. Metformin is one of the largest reported drugs to the US poison control centers with many serious outcomes and fatalities compared to any other oral anti-diabetic medicine [5]. Metabolic acidosis is the most serious adverse effect of biguanides overdose, including metformin [54]. The mechanism of metabolic acidosis associated with metformin inhibits mitochondrial complex-I of the electron transport chain in the mitochondria. Inhibition of complex-I causes a decrease in adenosine triphosphate, an increase in adenosine monophosphate, an overproduction of Reactive Oxygen Species (ROS), which contribute to metabolic acidosis. Besides, metformin exposure may cause (1) Inhibition of mitochondrial glycerophosphate dehydrogenase, (2) Blunting the conversion of glycerol-3-phosphate to Dihydroxyacetone phosphate, (3) Inhibiting gluconeogenesis from glycerol. The combination of these pathways finally contributes to metabolic acidosis [55]. Several studies have reported gastrointestinal symptoms associated with biguanide overdose, including nausea, vomiting, abdominal pain, diarrhea, and acidosis [56–59]. There is no clear explanation for how metformin overdose leads to gastrointestinal complications [60]. Although, it may be the result of increased intestinal glucose circulation, increased glucagon-like peptide-1, altered bile acid circulation, or altered intestinal bacterial flora [61].

Even though biguanides and sulfonylureas may be identified in urine analysis, this method is not commonly employed in facilities with limited resources. Our approach has the advantage of being based entirely on clinical presentation and laboratory data available in most healthcare systems. Considering this model, the patients exposed to antihyperglycemic agents would be diagnosed earlier. Because the prognosis for this type of exposure depends on how soon therapy is started. The most important point to remember is that since exposure to biguanides has a different complication than exposure to sulfonylureas, the management will differ. However, the clinical and laboratory findings are quite comparable; therefore, it is imperative to differentiate between them when making a diagnosis.

The strength of our study is that we use large-scale data from the National Poison Data System that help physicians diagnose biguanide and sulfonylurea overdoses accurately. However, some limitations should be taken into account. First, given that the case recording is based on self-report, the American Association of Poison Control Centers has found it difficult to confirm the exposures as poisoning. Second, there has been no information on the long-term outcomes of biguanides and sulfonylureas poisoning. Third, over-fitting is a common problem in machine learning models, especially for decision tree. We used 10-fold cross validation and also different max_dept and other hyper parameters tuning to minimize the overfitting risk. In order to increase predictive accuracy, future research should be conducted using novel biomarkers, larger datasets, improved data collection methods, and more sophisticated modeling methods. Furthermore, it is recommended that future studies in other settings use an external validation methodology through a separate cohort study in order to evaluate the generalizability of the model. Fourth, the NPDS database is not publicly available and it is recommended to evaluate the same approach with similar datasets which are openly/publicly available, for common features to validate the approach and reproducibility of the claimed findings.

## Conclusion

We successfully developed a different machine learning model as well as a decision tree-based approach with high accuracy to diagnose these two anti-diabetic poisonings. Physicians, can take advantage of this model and utilize it to early diagnose biguanides and sulfonylureas exposure because of its clear and concise interpretation and high accuracy. This model can help toxicology consultants in discriminating of biguanides and sulfonylureas poisonings. These machine learnig models could be improved in the future by applying the results of this study in generating practical applications or software, which can generate more features in clinical practice. Also the current algorithms should be tested prospectively in poison centers and clinical settings.

## Data Availability

The data that support the findings of this study is available from corresponding author but restrictions apply to the availability of this data, which is used under license for the current study, and so is not publicly available. The data is, however, available from the authors upon reasonable request and with permission of National Poison Data System (NPDS) administrator.

## Abbreviations

NPDS: National Poison Data System
AAPCC: American Association of Poison Control Centers
AUC: Area Under the Curve
CFR: Code of Federal Regulations
DT: Decision trees
